# The Uterus and the Human Ovum, during the First Months of Pregnancy

**Published:** 1833-04-01

**Authors:** 


### VIII.

**THE UTERUS AND THE HUMAN OVUM DURING THE FIRST MONTHS OF PREGNANCY, REPRESENTED ACCORDING TO NATURE. By Dr. *Burkhard William Seiler*, Physician to the Royal Court of Saxony, &c. &c. With Twelve Copper Plates. Dresden, 1832.**

IN the introduction, the author having paid the tribute of praise justly due to the works of Hunter and Soemmering, which he terms the ornaments of English and German literature, he apologizes for himself by saying, that the talent and researches of these two celebrated individuals have done so much for the subject on which they treat, that little remains for him but to fill up the gaps which they have left, not from any lack of industry or zeal, but rather of the necessary opportunity of examining a sufficient number of embryos and the impregnated uterus in perfectly healthy females, especially as regards their condition at the various periods of utero-gestation. He intimates that it is not sufficient to come to general conclusions from observations made on the uteri, *i. e.* obtained in a casual way, as circumstances permitted; but that in order to draw any really useful deductions, it is necessary, not only to devote oneself entirely to the subject with zeal and assiduity, but to obtain possession of a number of fresh embryos, taken from *perfectly healthy* females, and in a regular series, that the progress of the phenomena of Nature may be carefully and clearly traced; that such an opportunity falls to the lot of few, but that *he* has been so fortunate as to have the opportunity, and he hopes that he has made a good use of it.

He states that, for some years, Hunter's doctrines of the developments of the impregnated uterus were received almost without limitation, and were generally considered sufficient; but that he, not having described according to nature the "membrana decidua reflexa" at every period of gestation, gave rise to different views respecting their form, and even of their existence:—Moreover that, in later times, the important researches of Baer, Blundel, Breschet, Burns, Cruikshank, Home, Dutrochet, Meckel, Oken, Dollinger, Weber, Burdach, Jörg, Lobstein, Carus, Dumas, Pander, Prevost, Rathke, Velpeau, and others, have explained many circumstances, and shewn that the opinions previously entertained were weak, contradictory, and little grounded in facts; that it, therefore, became necessary to put them to the test of further experiment, by again referring to preparations which unfold, at all events, the chief moment of each particular development.

Speaking of the decidua, he says that some authors compare the decidua vera to the well-known membranous product of inflammation, an opinion to which he does not subscribe—that others describe the extremities of blood-vessels on the inner coat of the uterus, which, says he, cannot be demonstrated in preparations; that, even respecting the thickness of that coat, we are still at variance; that the existence of the membrana decidua reflexa is quite denied by some, whilst, according to others, this coat, in consequence of a pressing inwards of the decidua vera by the ovum, is formed, and thus gives rise to another opening or cavity; that this view of the matter does not owe its origin to the investigating of *healthy* specimens of ova during their situation in the womb, and that it is not grounded on observations made at the chief period of the development; that it has, however, led to the perfectly false adoption of a "membrana decidua primaria et seroteria."

That on the subject of the chorion, its layers and flakes, very various views are entertained, and even the almost forgotten opinion, of the immediate vascular communication and connexion between the mother and the fruit, has of late years found its defenders.

Respecting the uterine and the fœtal placenta there remains so much uncertainty, that the learned and shrewd naturalist, Baer, takes an opportunity, in one of his latest publications ("on the Vascular Connexion between the Mother and Child"), to set forth the supposition, that it may be in man as he has seen it in dogs, viz. the maternal and fœtal placenta grown together. No less wavering, he says, are the opinions entertained respecting the form and situation of the allantois in the human ovum. Even the already well-grounded view (as it seemed) of the connexion of the navel bladder with the intestines, met with opposition a few years ago, and it seemed, indeed, according to the investigations in animals, that some further notices on the subject were necessary.

The author then states, that accident has afforded him the opportunity, during a period of 12 years, of dissecting and examining 30 impregnated uteri, all of *perfectly healthy females*, and at different periods of utero-gestation; that some of them were injected—at one time the uterus or placenta alone, at another, both in the same preparation. In this way, he states that he has been able to furnish the museum of the Medico-Chirurgical Academy at Dresden with a series of preparations, which exhibit the principal moment of development during pregnancy, both in the uterus and the ovum, and whose numbers he proposes, in the second part of this work, to introduce as a note to the description. In order to obtain more information on this subject, he says he examined preparations taken from horses, dogs, swine, cows, and sheep, in which the time of fructification was observed as nearly as possible; that he compared the results he came to himself with the observations of others, and took into consideration also the passing phenomena of oviparous animals. He hopes, therefore, that he has been able, in this manner, to arrive at right conclusions, and to throw some light on a subject which has hitherto remained in so much doubt and obscurity.

This work, he says, will contain all that he has to say on the subject relative to man. The results of his observations on animals it is his intention to publish in parts, in the course of the Summer, under the title, "Investigations of some Parts of the Ova and Embryos of Animals." He proposes to treat particularly of the allantois and the navel bladder, the corpora Graafiana, &c. and other circumstances and peculiarities which he has himself observed, not only in dogs, swine and cows, but in sheep, the horse, and in man; he proposes, also, to set them forth in their proper order, as they appear in succession. The author seems to be aware that the subject is not without difficulties, and especially that in the inferior animals, the earliest periods are involved in much obscurity, the parts having then very much the appearance of jelly; and that, until the decidua reflexa is fully and fairly formed, the preparations are so tender and so easily torn, that it requires some experience to handle them, and to profit by the investigation. He wishes to institute comparisons between the phenomena which take place in man and in the brute, and so to illustrate and explain the great designs of Nature, as to render the work useful, not only to the teacher of anatomy and the physiologist, but also to those practising the obstetric art; and he hopes that his drawings and illustrations may, in some degree, make up for the want of the preparations themselves, which are not always at hand. The text of the present work is divided into two parts; the first contains a general explanation of the plates, and a sketch of the results of the author's observations on the uterus and ovum during the first months of pregnancy; the second part treats more fully of the author's opinions and the opinions of others, from remote periods up to the present time. Some of the drawings the author was pleased to lay before the Assembly of Naturalists and Physicians at Berlin, in the year 1828, and was so happy as to receive the praises of the meeting; among others, Professors Rudolphi and Tiedemann honoured him with their countenance. The author, therefore, recommends himself with confidence to the notice of the literary world, and asks only that liberality and impartial criticism which distinguish the true philosopher; he will then be encouraged to prosecute the subject he has undertaken, and to publish hereafter the results of his observations in the form of a supplement.

We think the author has done great justice to the subject he has taken in hand, and is worthy of public confidence; he seems to have set about the undertaking with great assiduity and in a scientific manner; he has availed himself of the opportunities afforded him, and should be encouraged to proceed. He begins in a tangible, proper manner, and throughout the work there is less of that soaring of the imagination in which the Germans, in the warmth and ardour of their temperament, are apt to indulge—we should rather have said there is none. We cannot charge the author with being speculative; he dwells, perhaps, a little more on the subject of the decidua, and the doctrines of Hunter and Bojanus, than may be necessary, as we do not see that his remarks are likely to lead to any practical result, or to establish the point at issue. They refer chiefly to the manner in which the deciduæ are formed; the "membrana decidua vera" of Hunter he declares to be—

"No new product, resulting from impregnation, but the loosened, and now vascular and highly organized, inner lining of the uterus itself, which has necessarily separated and richly developed itself. It is, when in a healthy state, of a red colour, and from one line to at most a line and a half thick, and it is only in aborted or diseased ova that we see it much thicker, as it is represented in the 8th table. In the first weeks of impregnation (how long, I cannot exactly determine), it is covered over with a white slime, just as the decidua reflexa has formed itself, and we are able to distinguish two perfect layers united together: the outer one loose, cellular, or mucous and vascular, and an inner one, at the cavity of the uterus, smooth and without vessels, which, at first sight, resembles a serous membrane, but which, on closer examination, appears to be very different, and more like a coagulated slimy membrane. This inner layer goes over the mouths of the Fallopian tubes, so that it entirely closes them, and in the more advanced periods of gestation, when the placenta is formed, and the membrana decidua reflexa has in part disappeared, is but imperfectly traced."

The "membrana decidua reflexa," the author states, he has represented "as it appears when the ovum is taken carefully out of its situation." He traces its beginning at the cut wall of the membrane of the uterus, and describes it as lying some lines wide, exactly on the outer layer of the membrana decidua vera, so that (he says) it forms the inner layer of the same, and entirely surrounds the ovum.

"That part of the decidua which lies free, closes the fore part of the cavity (as is represented in the fourth table), discloses small hollows, in which the shaggy flakes of the chorion rest, and by which the ovum at this period is made fast." 12.

That lastly, the os uteri is entirely closed by these membranes.

The author attempts to describe in regular order, as the phenomena of gestation proceed, the changes which take place as the uterus and its appendages develop themselves. Thus he speaks of, and illustrates in a very proper manner, the appearance of the ovaria and fallopian tubes, when impregnation has taken place, and the ova are descending into the uterus. Although the author deserves great credit for the manner in which he handles this part of the subject, one cannot help wishing that he had accompanied his illustrations (which we consider valuable) with more full remarks, as it is a subject of great importance as connected with jurisprudence, and one which every medical man is liable to be called to consider, and would do well to make himself familiar with. We are far from wishing to throw any blame on Dr. Seiler, for we think the scientific world are greatly indebted to him for what he has done; but, as he seems to have devoted so much time to the subject, and has had opportunities which do not fall to the lot of every one, the profession naturally look to him for information on this matter, and perhaps this hint may not be lost: if so, we shall be glad to have been the means of eliciting, from a man of talent and research, the elucidation of a subject which cannot fail to give Dr. Seiler additional claims to the public gratitude; and should he succeed by his exertions in saving only one life, by vindicating the character of an innocent, though suspected man, he will, we are sure, consider himself amply repaid. It is an awful thing, to reflect that we have been the means of condemning the innocent. In a court of law, the decision often depends entirely on the medical evidence; we are in duty bound to lean to the side of mercy, but justice requires that we speak truth. Though it be better that 10 offenders escape unpunished, rather than one just man be sacrificed, still, if religion teach us to reward virtue and punish vice, it is our duty to speak out, expose villainy if we can, and make of it a proper example. How many are led by their passions to commit crimes! How many have occasioned death unintentionally or ignorantly! How often do we hear of medicaments, and even poisons, being administered with a view to produce abortion! How many unfortunate females, fancying themselves with child when they were not, have been overcome by shame, and the dread of what they imagined, to destroy themselves by poison! and how often has the suspicion, in such cases, fallen on their lover, who, though innocent of the major offence, may have found it very difficult to clear himself! How much, then, depends, in such a case, on the medical evidence! The subject is one which is still involved in much obscurity; the appearance and condition of the organs of generation, with their appendages, before and after conception, are still imperfectly understood, and we are liable to be led by them into much error; and on the error which we commit depends, perhaps, the happiness and reputation, if not the life, of a worthy individual. It is the duty, therefore, of every lover of justice—every advocate of truth, to urge those who, like Dr. Seiler, have taken up this branch of the profession, to investigate it particularly in all its bearings, that, however scientific may be their physiological researches, they may not lose sight of the real end of all philosophical enquiry—the intellectual and physical happiness of mankind. We do hope, therefore, that Dr. Seiler will not lose sight of the practical inferences to be drawn from physiological observation, and that he will hereafter point out to us himself such improvements, and useful deductions, as his researches may lead to; his labours will then be doubly esteemed, and his works will not only be read by the curious and praised by the ingenious, but they will be appreciated by all who wish well to their fellow-creatures.

Little more need be said respecting Dr. Seiler's work. We think it may confidently be recommended to the notice of the profession; it is calculated to facilitate the study of the subject it is intended to teach; it is clearly written, and free from speculation or ambiguity, and will, therefore, be highly useful in the hands of the beginner. The plates are distinct and good, without being expensive, and are good illustrations of the text; they not only give us a clear idea of the progress of utero-gestation, and of the phenomena which occur as the healthy uterus develops itself, at the different periods, until it be relieved of its burden, but they explain to us the changes which the organs themselves undergo—the formation of the placenta and decidua—the position and condition of the ovum and fœtus, and also the nature of abortions during the early months, as far, at least, as is necessary for the object in view. The drawings, he says, were made from preparations, taken from naturally healthy females. The author gives us an excellent illustration of the formation and nature of the shaggy flakes of the chorion. In a word, we consider Dr. Seiler's work as a valuable acquisition to the medical world, and hope that it will meet with a good reception.

